# Optical push broom effect by a moving refractive index front in a silicon Bragg waveguide

**DOI:** 10.1038/s41598-026-36302-x

**Published:** 2026-01-23

**Authors:** Boyi Zhang, He Li, Xinlun Cai, Felix Vega, Juntao Li, Manfred Eich, Alexander Yu. Petrov, Mahmoud A. Gaafar

**Affiliations:** 1 Institute of Optical and Electronic Materials, Hamburg University of Technology, 21073 Hamburg, Germany; 2 State Key Laboratory of Optoelectronic Materials and Technology, Sun Yat-sen University, Guangzhou, 510275 China; 3 Directed Energy Research Centre, Technology Innovation Institute, SE45-01 Abu Dhabi, United Arab Emirates; 4 Institute of Functional Materials for Sustainability, Helmholtz-Zentrum Hereon, 21502 Geesthacht, Germany; 5 Department of Physics, Faculty of Science, Menoufia University, Menoufia, Egypt

**Keywords:** Optical push broom, Silicon waveguide, Signal trapping, Nonlinear pulse propagation, Optics and photonics, Physics

## Abstract

Here we report the first experimental demonstration of signal trapping by a refractive index front in a silicon waveguide with a periodic perturbation, the so-called optical push broom effect. The refractive index front generated by a fast pump pulse captures and confines the energy of a CW signal propagating with smaller group velocity near to the band gap of a Bragg grating introduced in the waveguide. The energy of the CW signal is shifted in frequency, accelerated to the front velocity and thus accumulated inside the front. In the experiment a 2 ps free carrier front was generated via two photon absorption of the pump in the silicon waveguide. It collects approximately a 30 ps long packet of the CW signal inside it. The presented effect can be utilized to compress signals in time and space.

**Supplementary Information:**

The online version contains supplementary material available at 10.1038/s41598-026-36302-x.

## Introduction

Dynamic frequency and bandwidth change of light is a highly desirable functionality in optical information processing^1,2^. Particularly interesting is the phenomenon of signal trapping by nonlinear pulses as this trapping increases the interaction time and thus the potentially achievable level of spatial/temporal compression. One of the prominent trapping mechanisms used in supercontinuum generation is provided by a decelerating soliton pulse, where a faster signal pulse coming from behind the soliton is trapped after interacting with the soliton^3–5^. In this case a signal that is reflected by the trailing edge of the soliton, is trapped and, similar to optical analogue of event horizon^6,7^, cannot leave the soliton, which is continuously slowing down. The pulse trapping in this case was explained by an effective ‘gravity-like’ potential that is produced by the decelerating soliton^4^.

On the other hand, the trapping can also be induced by a refractive index front moving with a constant velocity in a waveguide with hyperbolic dispersion^8,9^. The dispersion relation is shifted by the front and leads to an indirect transition of signal light where its frequency and wavenumber both are changed^10–16^. The indirect transition normally occurs to the state in the unperturbed waveguide or states of perturbed waveguide, leading to the reflection from^14,17,18^ or transmission through the index front^12,19,20^. However, in the special case of hyperbolic dispersion a slow light signal can be trapped inside a fast co-propagating front if it does not find any state in the waveguide before or after the front after the interaction, i.e. does not undergo either inter- or intraband transitions. Alternatively speaking the signal is accelerated up to the front velocity and further interaction does not lead to group velocity change. This effect, named as “optical push broom”, has been theoretically proposed by de Sterke^8,21^ and experimentally realized^9^ in a fiber Bragg grating. Such trapping also leads to the pulse compression as the energy of the input signal is concentrated in the front. For a finite signal pulse the optical push broom compression leads to a finite compression ratio^15^. In case of a very strong front, the dispersion relation is not only shifted but also significantly changes its slope^10,18,22–25^. In this case even in homogeneous media the signal light might not find states before and after the front is trapped. Continuous compression is expected in this case^26^.

For the optical push broom^9,13^ the pulse compression can be estimated from frequency shift in the front. The accumulated frequency shift of the signal $\Delta \omega$ is proportional to the time spend inside the front $\Delta t$ and front temporal slope^13^:

1 $${\Delta }\omega = \left( {\frac{{\partial \Delta \omega_{{\mathrm{D}}} }}{{\partial t_{{\mathrm{f}}} }}} \right){\Delta }t$$

where ${t}_{\mathrm{f}}$ is the front duration and ${\Delta \omega }_{\mathrm{D}}$ is the shift of the dispersion curve induced by the front. Therefore, different temporal parts of the signal automatically accumulate a linear frequency shift according to the time when they enter the front. This way, the signal time function is directly converted to frequency function, where the frequency distribution has a width equal to ${(\Delta \omega }_{\mathrm{D}}/{t}_{\mathrm{f}})\tau$, where $\tau$ is the signal pulse duration. As an example, for the dispersion shift ${\Delta \omega }_{\mathrm{D}}$ corresponding to 1 nm wavelength shift, front duration of ${t}_{\mathrm{f}}=1\text{ ps}$ and an initial signal duration of 30 ps the bandwidth of the shifted signal can approach 30 nm. This corresponds to pulse duration of 0.1 ps and the compression factor of 300.

The push broom experiment by Broderick et al.^9^ was limited by the low Kerr nonlinearity of the fiber and a weak perturbation of the Bragg grating. Compared to the fiber-based approach integration of the push broom effect to sub-millimeter lengths on a silicon chip has many advantages. Refractive index perturbations in the order of 0.003 can be obtained in silicon with pulsed lasers having several picosecond pulse duration and peak power in the order of 10 W^11,12,14^, compared to 10^6^ W required in fibers^9^. This low power induced perturbation would allow the switching of a stronger (compared to that of the silica fiber case) Bragg grating with a band gap opening in 1 nm range. Contrasting our work with the pioneering efforts of Broderick et al., our study leverages the high nonlinearity of Si waveguides to realize the push broom effect in a 1 mm waveguide, in contrast to 8 cm long fiber Bragg gratings. Another advantage of silicon Bragg grating is that we can easily control (engineer) the dispersion relation, by designing its geometric parameters^27^, by changing the 2D geometry of the waveguide, in contrast to fiber Bragg gratings, which are formed by writing a periodic variation of refractive index along the core of the fiber using an intense UV laser and where the refractive index contrast and resulting band gap are limited.

Here, we show experimentally for the first time the signal trapping inside a moving refractive index front in a silicon waveguide. We present the explanation of the trapping effect as an indirect photonic transition and discuss the conditions for required band gap opening and nonlinearity. With that we also identify an analogy to light stopping in tapered plasmonic waveguides^15,28–30^. The front is generated inside a silicon Bragg grating waveguide (SBGW) by two photon absorption (TPA) of a 2 ps long pump pulse at 1.55 µm wavelength with a peak power of 15 W which induces a free carrier (FC) density of ≈ 9·10^17^/cm^3^. The strong pump pulse tuned well away from the band gap of the Bragg grating traps wave packets of another co-propagating weak CW signal wave which is tuned near to the band gap. We show here, that in case of the trapping configuration 20 times more energy from the CW signal is converted to new frequencies, compared to the conventional cross phase modulation (surfing) configuration^31,32^—in which the signal and pump/front are moving with the same group velocity.

## Theory and approach

**Fig. 1:**
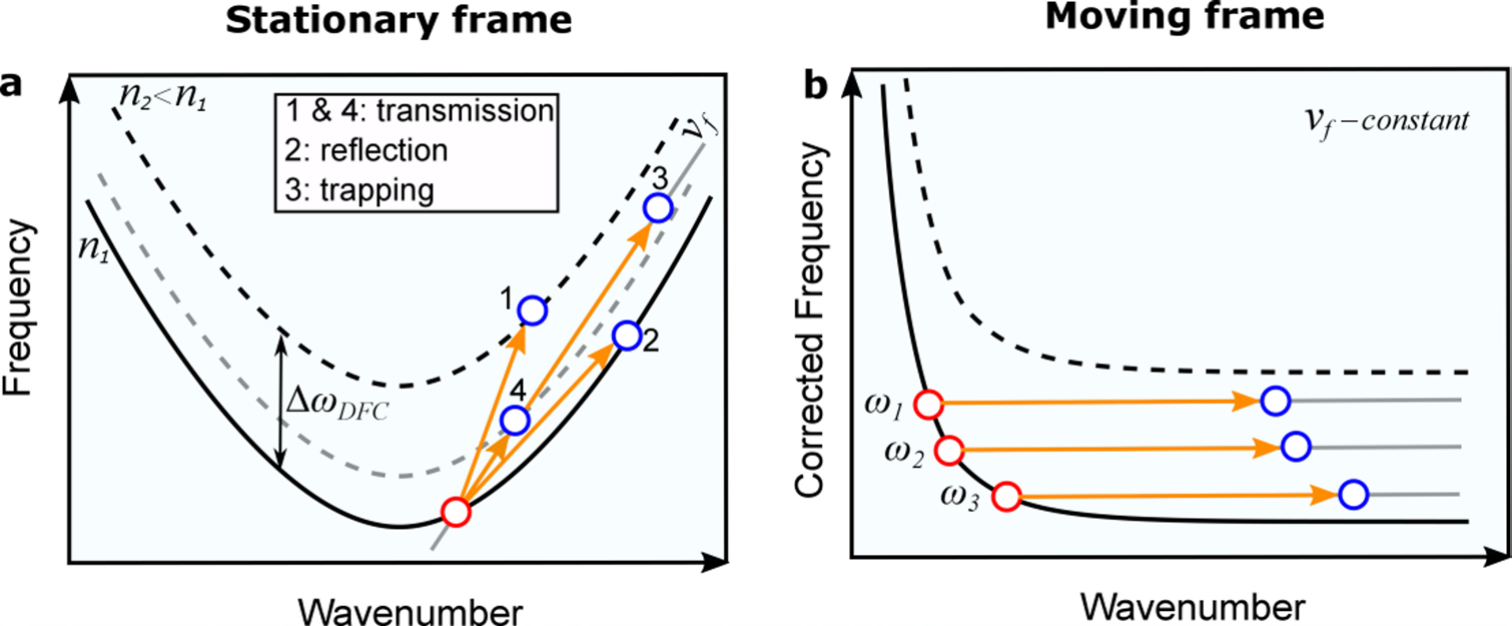
(**a**) Schematic representation in the stationary frame of different free-carrier-induced photonic transitions by changing the front velocity. The original band diagram is represented by the solid curve, while shifted band diagrams represented by the dashed black (strong index change) and dashed grey (weak index change). Red and blue circles indicate the initial and final state of signal wave, respectively. 1 and 4: transmission through the front, 2: reflection from the front and 3: signal trapping inside the front. (**b**) Schematic representation of the signal trapping (transition 3 in (**a**)) represented in the frame moving with the front. When different frequency components (${\omega }_{1}, {\omega }_{2}, {\omega }_{3}$) enter the index front they at the beginning will leave the original dispersion curve and will approach a constant positon between original and shifted dispersion curves, which corresponds to a certain position inside the front.

Interaction of light signal with an index front leads to an indirect photonic transition with a simultaneous change of its frequency $\Delta \omega$ and wavenumber $\Delta \beta$^10,13^. The direction of the indirect transition in the band diagram is defined by the phase continuity at the front, where $\Delta \omega /\Delta \beta$ is equal to the front velocity ${v}_{\mathrm{f}}$^11,14,33^. Therefore, the angle of the indirect transition induced by the front is defined by the front velocity. Figure 1a shows a schematic representation of four different indirect transitions in a waveguide with hyperbolic dispersion. In particular, the hyperbolic dispersion curve converges to a straight line at large wavenumbers. We consider here an example of a negative index front due to the effect of FCs generation. Thus, solid and dashed curves correspond to the dispersion relations of the system before and after the index front, respectively. In this schematic, the front velocity (indicated by the slope of the orange arrow) and the group velocity of the signal are co-directed for all transitions. The straight grey line represents the phase continuity line with a slope equal to the front velocity (we show only one line for clarity). The red and blue circles indicate the initial and final states of the signal wave, respectively.

Several transitions are shown depending on the front velocity. Transition 1 corresponds to signal transmission through the front, i.e. the final state of the signal after interacting with the front is behind the front (i.e. inter-band transition)^11^. Transition 2 describes the situation when the signal pulse does not find state behind the front and therefore reflects from it in the forward direction (i.e. intraband transition)^14^. However, if the phase continuity line does not cut either the unperturbed (solid black) or the completely perturbed (dashed black) dispersion curves, the signal will be trapped inside the index front, as it will not find any state inside the waveguide before or after the front (transition 3). For the realization of the trapping effect, the front-induced band diagram shift ${\Delta \omega }_{DFC}$ should be sufficient, otherwise the signal will transmit through the front (transition 4 in Fig. 1a). For example, to trap a signal located initially exactly at the band edge, the necessary band diagram shift should be ${\Delta \omega }_{\mathrm{DFC}}>{\Delta \omega }_{\mathrm{PBG}}/2$, where ${\Delta \omega }_{\mathrm{PBG}}$ is the photonic band gap (PBG) opening. In other words, a phase continuity line drawn from the point at the band edge is offset to the asymptotic branches of hyperbolic dispersion relation by half the band gap. A smaller band diagram shift would be required if the initial signal wavelength is not at the band edge. Insufficient shift might result in transmission through the front (transition 4). We reduce the required shift by locating the signal slightly away from the band edge.

The transition can be also discussed in the frame moving with the front (Fig. 1b)^13^. In this frame the front is a stationary perturbation and thus there is no frequency shift of the signal. An interesting fact is, that in this frame, the corrected dispersion relation $\omega {\prime}\left(\beta \right)=\omega \left(\beta \right)-{v}_{\mathrm{f}}\cdot (\beta -{\beta }_{0})$ in case of trapping configuration is represented by a hyperbolic function with one of the asymptotic branches parallel to the wavenumber axis with zero group velocity at infinite wavenumber $\beta$ (See Fig. 1b). It reminds of the dispersion relation of a surface plasmon polariton^15,34^. When a surface plasmon polariton encounters a tapered perturbation that shifts the dispersion curve and does not allow further propagation then it is not reflected but is trapped at the transition leading to nanofocusing effects^28–30^. Different frequency components will freeze at different positions in the taper, leading to so-called “trapped rainbow”^27^. Exactly the same is obtained now with a moving front in a waveguide with a hyperbolic dispersion curve $\omega \left(\beta \right)$. In the frame moving with the front the signal enters the stationary perturbation and stops inside the front without reflection. A monochromatic CW signal will approach zero group velocity in respect to the front and will stop at a defined location inside the front, thus ultimate compression would be achieved. In a real situation an input signal has a finite duration and thus a certain frequency distribution. The different spectral components will stop at slightly different locations inside the front.

**Fig. 2:**
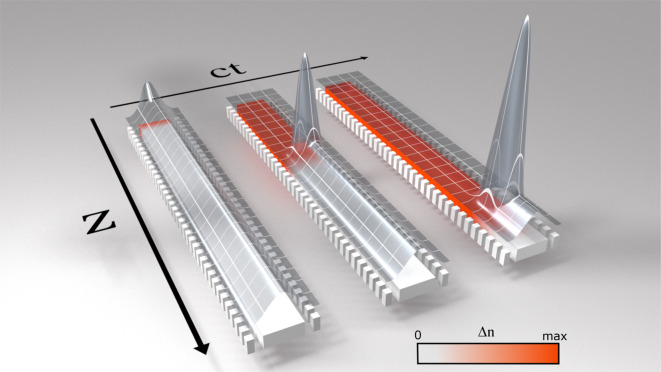
Schematic of the trapping effect inside a SBGW. A free carrier front propagates with the velocity of the pump pulse (red color). The fast front will frequency shift the slow signal wave and accelerate it to move with the front. The signal intensity is presented by a 3D surface, it accumulates at the front with time. The initial signal energy is swept out of the waveguide and no signal is present behind the front.

The basic concept of trapping effect inside a SBGW is visualized in Fig. 2. Such waveguide has a hyperbolic dispersion relation, which appears in weakly perturbated periodic structures, such as Bragg stacks^35^, fiber Bragg gratings^36,37^, silicon Bragg gratings^38,39^ and waveguides with periodical corrugation^40,41^. We use SBWG with periodic silicon wings. We will conduct our experiment using a CW signal light and a pulsed pump similar to other work on pump probe interactions^6,7,9^. In Fig. 2, white color indicates the silicon material. A CW signal is confined inside the waveguide and propagates with small group velocity. When a pump pulse is launched into the structure, it generates FCs in the silicon waveguide and, consequently, induces a change of refractive index ${\Delta n}_{\mathrm{FC}}$ which propagates with the velocity of the pump pulse ${v}_{\mathrm{f}}$. The gradient of the induced index front corresponds to the pump pulse duration. The white and red colors represent the waveguide before the pump pulse (with refractive index ${n}_{1}$) and after the propagation of the pump pulse (with reduced refractive index ${n}_{2}<{n}_{1}$), respectively. The signal will be trapped at the front. As time progresses, the index front traps ever greater portion of the signal, so that ultimately almost all of the initial signal energy is swept out of the waveguide.

**Fig. 3:**
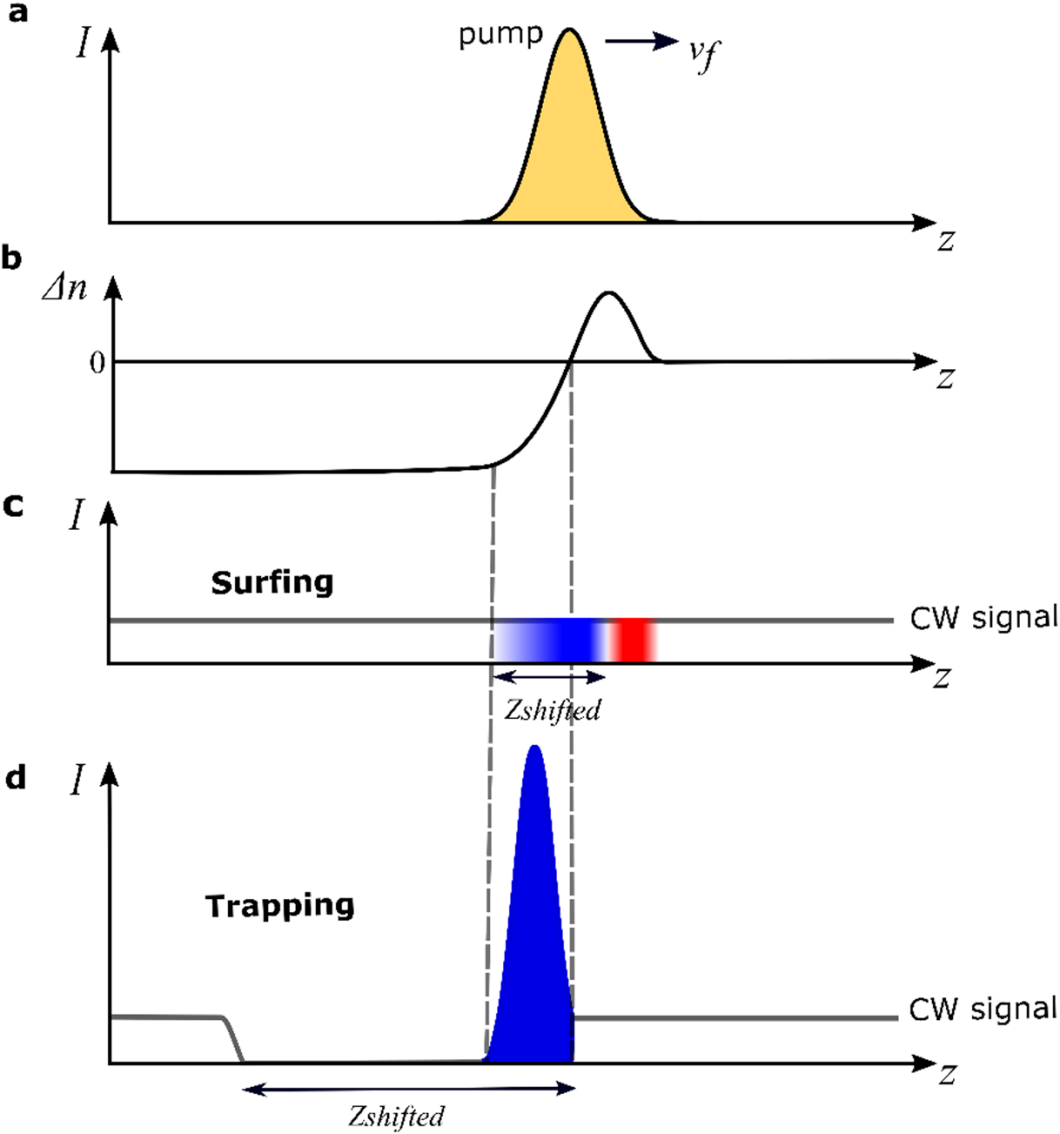
(**a**) Schematic of the spatial profile of the pump pulse at a given point of time, (**b**) the corresponding spatial change of the waveguide’s refractive index induced by the pump pulse, due to both Kerr effect and FCs injections (see Supplementary Note 4), (**c**) and (**d**) Schematic representations of the CW signal wave (its power is represented by grey solid line) shifted to new frequency, in case of surfing and trapping, respectively. The length ${z}_{\mathrm{shifted}}$ marks the length of the CW wave which is shifted to new frequencies. In case of surfing, the length of the shifted signal is limited by pump pulse size. Red and blue colors indicate the red and blue frequency shifted wave packets due to positive and negative slopes of the refractive index change, respectively. In case of front induced trapping, the shifted length can be much larger than pump pulse. Blue color again indicates the blue frequency shifted signal. The red frequency shift is negligible in this case as the fast front first red shifts the signal and then strongly blue shifts it.

Figure 3 illustrates the advantage of the front induced signal trapping to shift large portions of signal wave in one step, compared to the normal cross-phase modulation effect, which we call surfing here. Figure 3a and b represent a schematic of the spatial profile of the pump pulse at a given point of time and the corresponding spatial change of the waveguide’s refractive index induced by the pump pulse, assuming picosecond pump pulses, respectively (see Supplementary Note 4)^31^. Surfing occurs when the front propagates at the same speed as the signal and dispersion relation is a straight line, while the push-broom effect arises when the front moves faster than the signal and dispersion is hyperbolic. Surfing leads to positive and negative frequency shift due to positive and negative slopes of the refractive index perturbation, which result from the combined effect of Kerr nonlinearity and FC dispersion induced by the pump pulse in silicon^31,32^. In this case, the time span of the signal wave shifted to new frequencies is limited the by pump pulse duration, as we can see in Fig. 3c, where ${t}_{\mathrm{shifted}}$ is related to ${z}_{\mathrm{shifted}}$ as ${t}_{\mathrm{shifted}}=\left({n}_{\mathrm{g}}^{\mathrm{s}}\cdot {z}_{\mathrm{shifted}}/c\right)$, where ${n}_{\mathrm{g}}^{\mathrm{s}}$ is the group velocity of the signal. Here, we consider a CW signal wave with constant power over time (solid grey line). Red and blue colors indicate the red and blue frequency shifted wave packets due to positive and negative slopes of the refractive index change, respectively. We have to mention, that also in case of four wave mixing, the time span of the signal wave shifted to new frequency is limited by the pump pulse duration^42,43^. On the other hand, in case of the front induced signal trapping, or push broom effect, the time span depends on both the group velocity differences between the pump ${n}_{\mathrm{g}}^{\mathrm{f}}$ and the signal ${n}_{\mathrm{g}}^{\mathrm{s}}$ and on the interaction length $L$^44^:

2 $$t_{{{\mathrm{shifted}}}} = L\left( {\frac{{\left| {n_{{\mathrm{g}}}^{{\mathrm{s}}} - n_{{\mathrm{g}}}^{{\mathrm{f}}} } \right|}}{c}} \right)$$

Thus, in this case we can envisage shifting large portions of CW signal in one step (see Fig. 3d). We should mention that Fig. 3d is in the energy conserving approximation, thus the energy missing in the CW part is collected in the peak. However, in case of nonlinear losses the peak power will reduce. Overall, trapping leads to larger energy shifted to new frequency, and thus can be differentiated from surfing already in the spectral response.

The complex front function with positive and negative refractive index change is not a problem for the discussed trapping effect. The signal approached by the front is then first decelerated and red shifted by the positive slope of the Kerr effect and then accelerated and blue shifted by the strong negative slope of the FC effect. Finally, the signal is trapped in the negative slope. We also provide ray tracing simulation of the signal wave packets interacting with the front presented in Fig. 3b (see Supplementary Note 3). It can be seen there that all wave packets of the CW signal are trapped in the same position inside the front.

## Experiments

### Design and manufacture of silicon Bragg grating waveguide

**Fig. 4:**
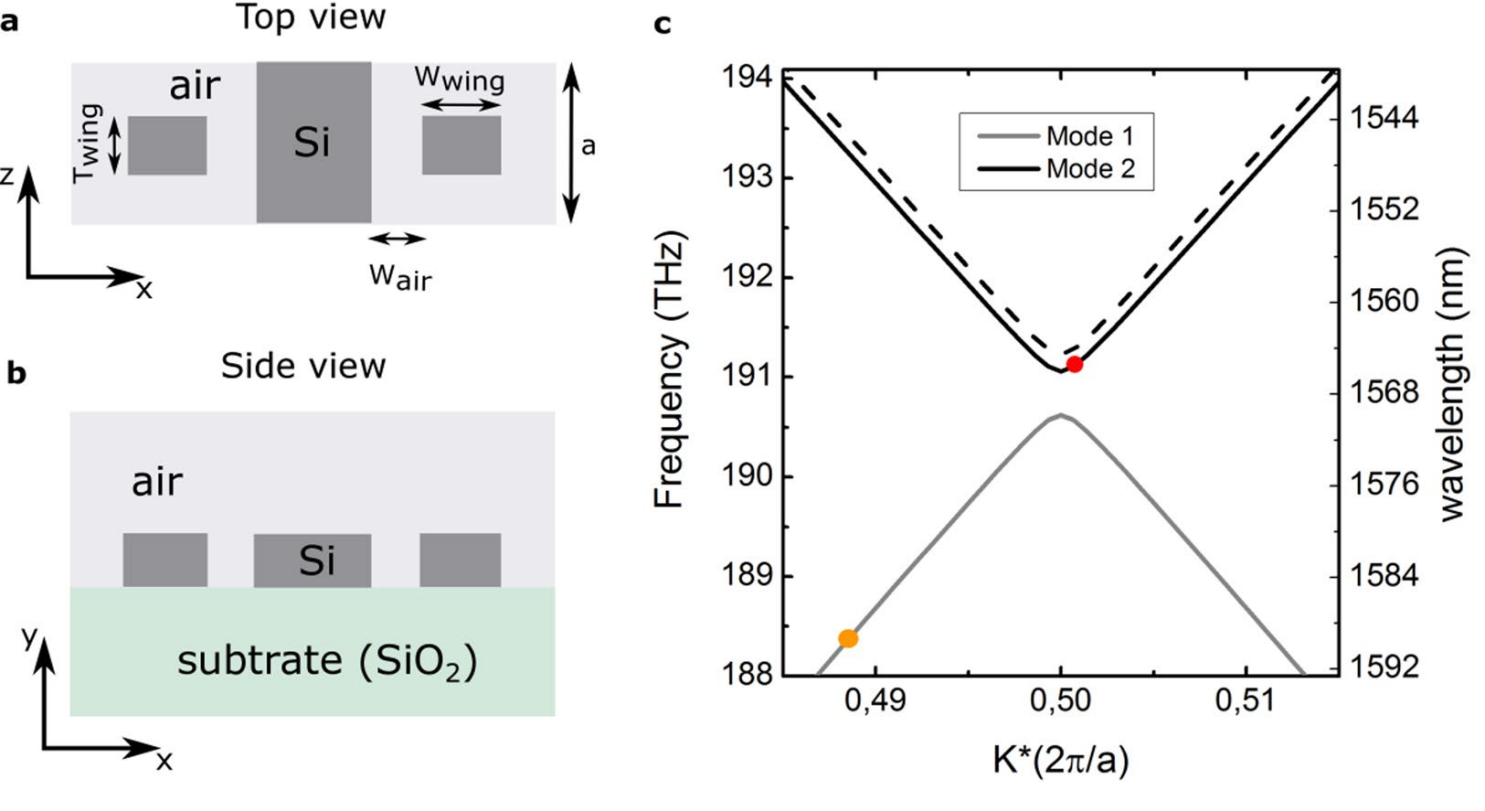
Schematic of the initial design of SBGW. (**a**) Top view of the Bragg grating. (**b**) Side view. The silicon slab height and width are 220 nm and 500 nm, respectively. The height, width (${W}_{\mathrm{wing}}$), and the thickness ${T}_{\mathrm{wing}}$ of the silicon wings are 220 nm, 220 nm and 150 nm, respectively. The air gap between the silicon waveguide and the wings is ${W}_{\mathrm{air}}=40 \mathrm{nm}$, and the lattice constant $\mathrm{a}$ is 330 nm. (**c**) The band diagram with two hyperbolic band, simulated from the listed parameters by CST eigenmode solver. Orange and red circles indicate the proposed pump and signal frequencies, respectively.

In order to satisfy the trapping requirement, a hyperbolic dispersion relation was designed by engineering the silicon waveguide with periodic wings similarly to Refs.^40,41^. The waveguide is implemented on a standard silicon-on-insulator (SOI) platform with a slab thickness of 220 nm and width of 500 nm. A periodic wing structure is then introduced as a weak perturbation to realize the desired hyperbolic dispersion relation. The initial wing structure was generated by CST Studio Suite as shown in Fig. 4a (top view of the structure) and 4(b) (side view), where dark and light grey regions denote silicon and air, respectively. The height, width, and the thickness of the silicon wings (ribs) are 220 nm, 220 nm and 150 nm, respectively. The air gap between the silicon waveguide and the wings is ${W}_{air}=40 nm$, and the lattice constant $\mathrm{a}\approx 330\text{ nm}$.

Figure 4c presents the simulated band diagram using the CST eigenmode solver. Periodic boundary conditions are applied along the propagation direction, while electric boundary conditions are imposed on the remaining boundaries, placed 660 nm away from the waveguide core. All boundary settings follow the default CST configuration. An adaptive tetrahedral mesh with a minimum edge length of 2.3 nm was employed to ensure numerical convergence. Solid black and grey lines represent the upper and lower branches of the dispersion relation, respectively, while the dashed black line represents the shifted band due to the presence of the FC front. Technically, if we positioned both the pump pulse and the signal wave on the upper band of the dispersion relation, it would be challenging to detect the blue shifted signal due to overlap with the pump. Therefore, we positioned the pump frequency on the nondispersive part of the lower band of the dispersion relation (orange circle).

Fabrication tolerances, such as variations in feature size and spacing, primarily lead to shifts in the bandgap wavelength and modifications of the bandgap width. To account for this deviation, a series of waveguides with incrementally varied geometric parameters were fabricated. Transmission and group index measurements were then performed to experimentally identify the waveguide, whose dispersion best satisfies the trapping requirements. As a result, the final device may differ from the nominal design while providing the desired dispersion profile. It should be emphasized that the trapping experiment relies on the resulting dispersion relation of the waveguide, rather than on any specific geometric parameters. Besides, different Bragg waveguide designs or different weak periodic perturbations, can give rise to similar hyperbolic dispersion characteristics.

**Fig. 5:**
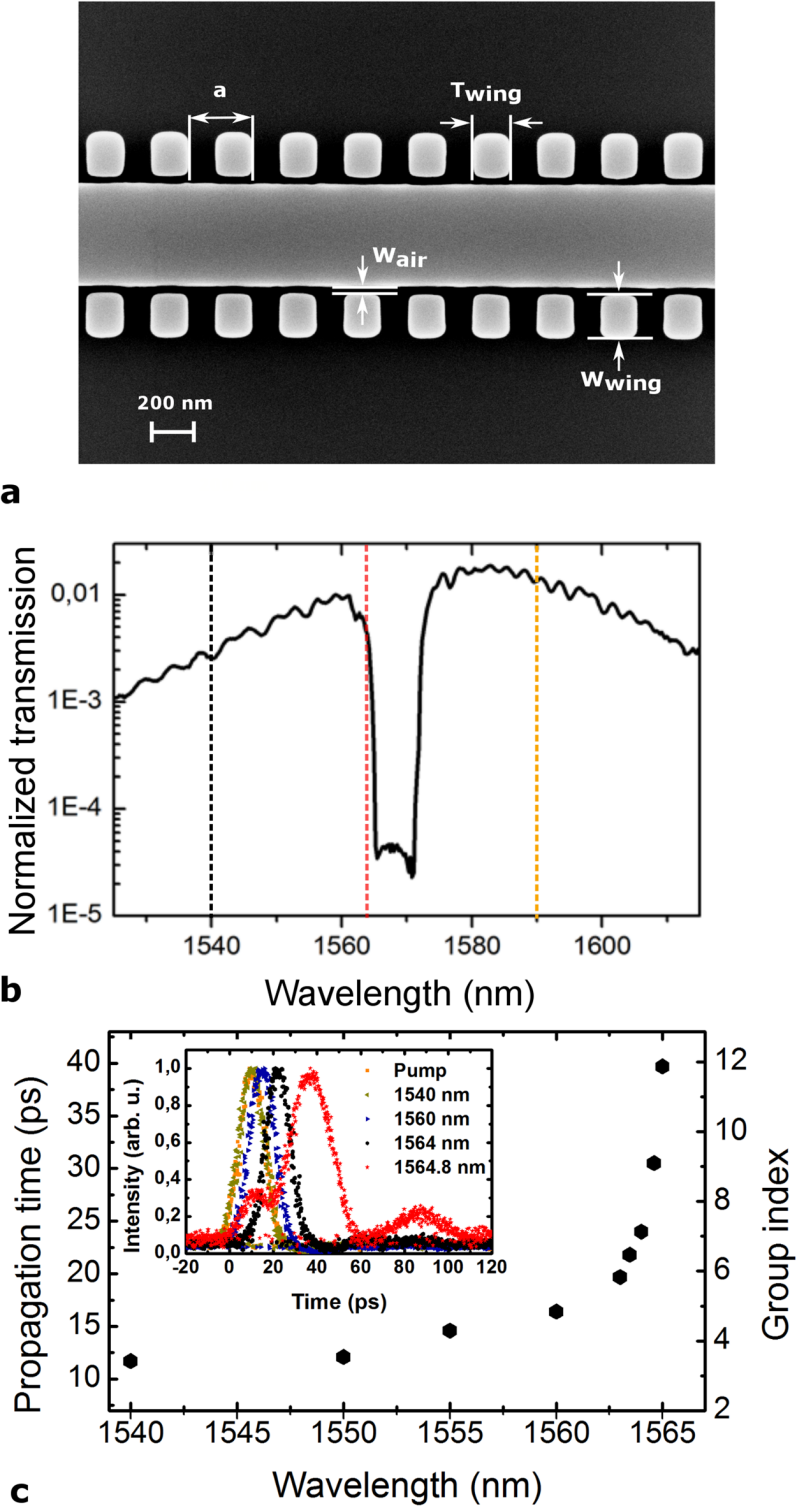
Characteristics of the selected SBGW. (**a**) SEM of the device. (**b**) Linear transmission. Black and red dashed lines indicate the wavelengths of the signal wave in case of surfing (1540 nm) and trapping (1564.8 nm), respectively. The orange dashed line indicates the wavelength of the pump pulses. Note that the bandgap deviates from the CST simulation. (**c**) The time delay and group index measurements. Inset shows the pulse temporal profiles, as the pulse approaches the band edge the third order dispersion distorts the pulse shape.

The SEM image of the final selected SBGW is shown in Fig. 5a. The corresponding measured linear transmission (including coupling loss) and the group index of the TE-mode of the 1 mm SBGW are shown in Fig. 5b and c, respectively. The broadband shape of the transmission curve is defined by the transmission characteristics of the grating coupler. For delay (group index) measurements, the transmission of a weak pulse with ≈ 0.4 nm bandwidth through the waveguide is measured by an optical sampling oscilloscope. As we can see, the group index increases as the pulse approaches the band edge, i.e. the light slows down. As noted above, the experimental bandgap wavelength and band width deviate from those simulated in Fig. 4c. Accordingly, all subsequent experiments and discussion rely on this measured dispersion, not on the exact geometry parameters from the initial CST design.

Finally, two grating couplers are used to couple the light into the SBGW. The grating coupler is 20 µm long and 12 µm wide. The grating consists of air hole rows in silicon^45,46^. This coupler has a 3 dB bandwidth of 30 nm with a peak coupling wavelength of 1570 nm, and a minimal coupling loss of $\approx$ 7 dB per coupler when the coupling angle is 13 $^\circ$. More details about the design and manufacturing of the waveguide and couplers are given in Supplementary Note 1.

### Pump-probe experiment set up

**Fig. 6:**
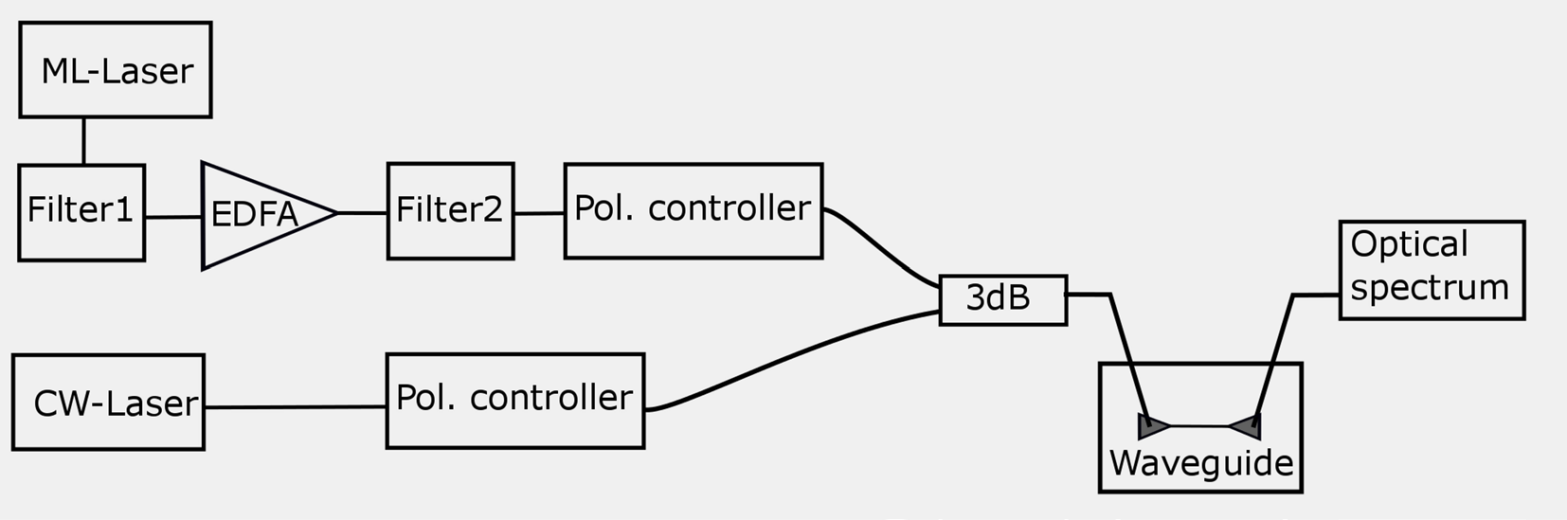
Schematic of the experimental setup. EDFA, erbium-doped fiber amplifier; Pol., polarizer and 3 dB, 50:50 beam combiner. All solid lines represent fiber coupling.

The pump probe setup is arranged as displayed in Fig. 6. A mode-locked fiber laser (Menlo system) with a 100 MHz repetition rate delivers pulses of approx. 100 fs duration with a wide spectrum ranging from 1500 nm to 1610 nm. The output is connected to a grating filter (Filter 1) with adjustable bandwidth and center frequency followed by an erbium-doped fiber amplifier (EDFA). The output after EDFA is connected again to a second grating filter (Filter 2) to suppress the noise at the signal wavelength. Pulses with tunable center wavelengths between 1525 nm and 1600 nm and bandwidths between 0.1 nm and 4 nm (33 ps–2 ps) can be produced with this configuration. The signal light is derived from a tunable diode laser (Photonetics PRI). It delivers up to 8 mW of CW light, tunable from 1500 to 1600 nm. We excite a quasi-TE mode of the waveguide with polarization predominantly oriented parallel to the chip surface. The grating couplers are also designed for the excitation of TE mode. The laser output is polarized, thus, to optimize the coupling efficiency from fiber in the grating coupler we employed separate polarization controllers for signal and pump. The pump pulse is subsequently combined with the CW signal light through a 50/50 (3 dB) beam combiner, which is then fiber-coupled to the Bragg grating using grating coupler. The propagation loss is estimated to be on the order of 10 dB/cm for Bragg-grating waveguide^47^, which is sufficiently small to be neglected over the 1-mm length of our waveguide and will be omitted to simplify the discussion. The optical spectra are measured by the optical spectrum analyzer (Ando AQ6317).

In our pump-probe experiments we consider two situations, cf. Fig. 3. The first case is when the signal wave and index front, are moving with the same group velocities, i.e. the signal surfing on the front. The other case when the signal wave is excited at the band edge with group velocity smaller than the front velocity and is accelerated and trapped by the approaching pump pulse. Black and red dashed lines in Fig. 5b indicate the wavelengths of the signal wave in case of surfing (1540 nm) and trapping (1564.8 nm), respectively. As seen from Fig. 5c, the pump pulse at ≈ 1590 nm has a group velocity matched wavelength at 1540 nm (${n}_{\mathrm{g}}^{\mathrm{f}}={n}_{\mathrm{g}}^{\mathrm{ss}}=3.5$), while the group index of the signal wave at wavelength of 1564.8 nm is ${n}_{\mathrm{g}}^{\mathrm{st}}\approx 12$. Far from the bandgap the dispersion relation of the SBGW converges to that of the standard waveguide which still has some dispersion and there is small group velocity mismatch between signal at 1540 nm and pump at 1590 nm. But we neglect the effect of this mismatch in our 1 mm waveguides as accumulated time delay between two waves is approx. 0.3 ps which is much smaller than the pulse duration.

We have to mention also, that Kerr-induced self-phase-modulation causes a temporally varying instantaneous frequency of the pump. This way, an initial non-chirped pump pulse acquires a frequency chirp. Still this frequency chirp does not change the pulse envelope, thus, the front shape as the pump is propagating at the frequency with negligible dispersion.

### Signal surfing and trapping inside the front

2 ps long pump pulses with 100 MHz repetition and 100 mW average power derived from mode-locked laser are launched into a 1 mm-long SBGW at a center wavelength of 1590 nm with a group index of ${n}_{\mathrm{g}}^{\mathrm{f}}=3.5$ (corresponding to orange dashed lines in Fig. 5b). We also feed in the low power CW signal, which co-propagates with the index front in the waveguide. For the surfing case, the signal wavelength is 1540 nm with a group index of ${n}_{\mathrm{g}}^{\mathrm{ss}}=3.5$ (corresponding to black dashed lines in Fig. 5b), while for the trapping case, the signal wavelength is 1564.8 nm with a group index of ${n}_{\mathrm{g}}^{\mathrm{st}}=12$ (corresponding to red dashed lines in Fig. 5b).

**Fig. 7:**
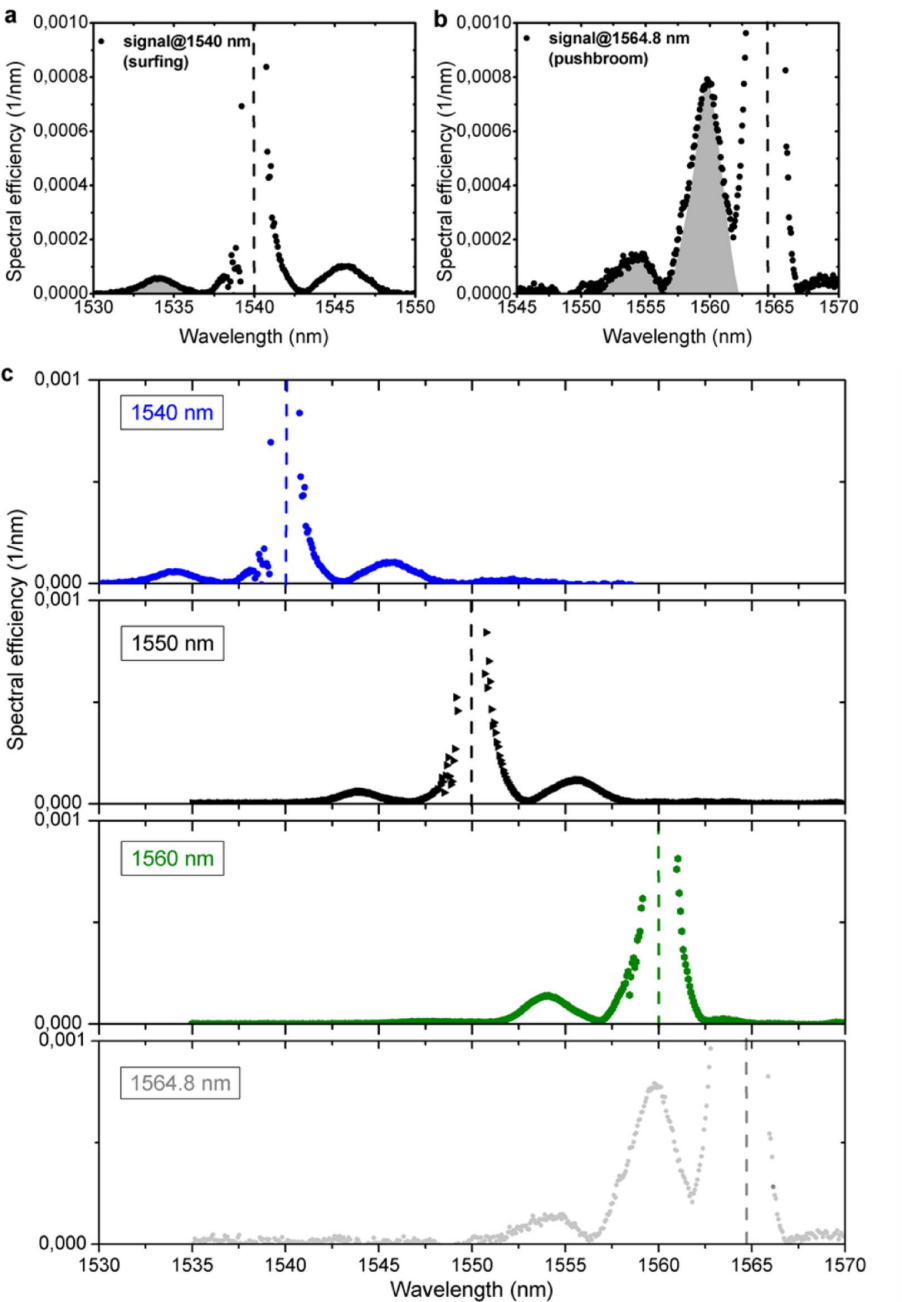
Experimental spectra recorded at the output of the 1 mm-long SBGW in case of (**a**) surfing (signal wavelength at 1540 nm) and (**b**) trapping (signal wavelength at 1564.8 nm). Dashed lines represent the center wavelengths of the initial CW signals. Shown is the spectrum of the CW signal that has been frequency shifted. The spectral efficiency is defined as the power per nm divided by the transmitted CW signal power. (**c**) Experimental output spectra for different input signal wavelengths.

Figure 7 shows the experimental output spectra of the frequency shifted signal normalized by the output CW power of the transmitted signal for both surfing (Fig. 7a) and trapping (Fig. 7b) situations for input pump peak power of 15 W. The corresponding CW signal powers at output without pump were ≈ 4 μW and ≈ 2 μW, respectively. The spectral efficiency is defined as the power per wavelength divided by the power of the output CW signal. Traces with and without pump pulses present in the waveguide are recorded and subtracted, leading to the shifted signal displayed on a linear scale (see Supplementary Note 2). The center wavelengths of the initial signals are marked by dashed lines. As we can see, clear peaks appear on both blue and red sides of the input signal wavelengths in case of surfing, due to the positive and negative slope of the pump pulse. The trapping shows much larger conversion efficiency and much less red shifted signal (see also Supplementary Note 2). There is also large power density observed around original CW frequency after the subtraction of the signal without pump pulse. These are frequency components corresponding to the temporal ‘shadow’ left inside the CW signal after the cut-out of the wave packets interacting with the front and wave packets that cannot enter the Bragg grating while the free carriers are there.

Figure 7c represents the recorded spectra at the output of the waveguide for different input signal wavelengths. As we can see, as the signal approaches the band gap more energy is converted by the co-propagating index front.

## Discussion

As we use a CW signal, only a small fraction of the total signal light can be converted within the finite length of the waveguide. The fraction η of the CW signal wave power that enters the front and is frequency converted including the FCs absorption is:

3 $$\eta = \eta_{{{\mathrm{CW}}}} \cdot \eta_{{{\mathrm{loss}}}}$$

where ${\eta }_{\mathrm{CW}}$ is the fraction of the CW signal power that is frequency converted, given by:

4 $$\eta_{CW} = \nu_{rep} \cdot \left[ {\left( {\frac{{n_{g}^{s} - n_{g}^{f} }}{c}} \right) \cdot L + t_{p} } \right]$$

where ${\nu }_{\mathrm{rep}}$ is the repetition rate of the pump pulses, $c$ is the velocity of light in vacuum, $L$ is the length of the waveguide and ${t}_{\mathrm{p}}$ is the pump pulse duration. The time span of the CW signal that was reached by the front within the waveguide length *L* is denoted by the first term in the bracket, while the second term relates to the CW signal wave that entered the waveguide together with the front for duration ${t}_{p}$. The CW signal coming later is reflected by the band gap of the dispersion relation shifted by the pump.

While ${\eta }_{loss}$ is a factor considering the absorption of the signal wave packets by FCs during their interaction with the plasma front, and is given by:

5 $$\eta_{loss} = \frac{{\mathop \smallint \nolimits_{0}^{L} e^{ - \alpha l} \cdot dl}}{L}$$

where $\alpha$ is the FC absorption coefficient. For $\alpha =30 {\mathrm{cm}}^{-1}$ at FC concentration of ≈ 9.5·10^17^/cm^3^^48^ (see Supplementary Note 4) and L = 1 mm, this leads to estimated ${\eta }_{\mathrm{loss}}\approx 30\%$.

We estimate the rise time of the negative front as $\tau =1.9\,\mathrm{ps}$ (see Supplementary Note 4) which is comparable to the duration of the pump pulse. In case of surfing (${n}_{\mathrm{g}}^{\mathrm{s}}=3.5$), and for ${\upnu }_{\mathrm{rep}}=100\text{ MHz}$, ${n}_{\mathrm{g}}^{\mathrm{f}}=3.5$, and $L=1\text{ mm}$, the maximal blue shifted fraction including the FC absorption of the incoming CW signal power due to the interaction with single pump pulses arriving at 100 MHz repetition rate is 0.57·10^−4^. While in case of trapping (${n}_{\mathrm{g}}^{\mathrm{s}}=12$), the expected maximally transformable fraction is 0.95·10^−3^. Thus, trapping should be 17 times more efficient than surfing.

From the measurements shown in Fig. 7, by integrating the area under blue shifted peaks for both surfing and trapping cases (marked by grey colors), we can see that the experimental blue-shifted conversion efficiency due to trapping is ≈ 0.71·10^−3^ while due to surfing is ≈ 0.35·10^−4^. Thus, the experimental conversion efficiency due to trapping is approx. 20 times higher than in the surfing case, which fits the analytical estimation and shows the advantage of the push broom effect. We measure conversion efficiencies that are 75% in case of trapping and 60% in case of surfing of what we expect from the theoretical estimation including the FCs absorption. The deviation can be attributed to inaccuracies in the estimation of the free carrier concentration and corresponding free carrier absorption coefficient as well as additional contribution of two photon absorption by signal and pump.

It should be also mentioned that since a CW signal was used here, only ${\eta }_{\mathrm{CW}}\hspace{0.17em}$= 0.02% (in case of surfing) and 0.3% (in case of trapping) of the CW wave power was participating in the interaction process, and this value then represents the 100% conversion efficiency. As all power of the CW signal at times between the pump pulses is not converted, thus, a small apparent conversion efficiency is observed. When taking into account only those fractions of the CW signal present at times when the interaction with the front takes place, the effective conversion efficiency of the signal wave packets trapped by the front is much larger and amounts to ≈ 25%. For signal pulses or pulse trains that have durations equal to the interaction time with the FCs front- instead of CW signal, we expect the conversion efficiency to approach this number. The CW signal used here is a practical way for studying frequency conversion as it has narrow spectrum and does not require the adjustment of the delay between pump and signal. It was also used previously for other front-induced interactions^6,14^.

In case of trapping the converted spectrum has a second peak at 1554 nm. It can be attributed to the CW wave packets which enter waveguide together with the front, and therefore have the largest wavelength shift. We can estimate the maximum possible wavelength shift of the signal in the structure $\Delta \lambda =\Delta {\lambda }_{\mathrm{D}}\cdot\Delta t/\tau$, by assuming the maximal time spent inside the front $\Delta t$ equal to the travelling time of the pump inside the 1 mm structure which is ≈ 12 ps, where $\Delta {\lambda }_{\mathrm{D}}=2.2\,\mathrm{nm}$, and $\tau =1.9\,\mathrm{ps}$ (see Supplemental Note 4). This yields to a maximum expected possible shift of ≈ − 13 nm, which approximately fits to the maximal obtained experimental shift of ≈ − 11 nm. The dip between the two peaks at 1556 nm might appear due to interference of the signal entering with the front und reached by the front in the waveguide. For longer waveguides, the trapping effect increases the time the signal spends inside the front—provided that the front strength, and thus $\Delta {\lambda }_{\mathrm{D}}$, is maintained—leading to a larger wavelength shift of the signal. A similar enhancement occurs with sharper fronts. In the case of a CW signal, and according to Eq. (4), the conversion efficiency increases with higher repetition rates, up to values below the inverse of the free-carrier lifetime.We have to mention, that in the case of signal reflection from a copropagating index front reported before^14^, the phase continuity line cuts through the unshifted band, thus the signal is reflected and stays in the waveguide before the front. We observe here, in case of push broom effect, 3 times larger CW conversion efficiency compared to the reflection effect^14^ even though the pump in the push broom is fast and spends less time in the waveguide.

## Conclusions

In conclusion, we have experimentally demonstrated a signal wave trapping inside a moving refractive index front propagating in a 1mm long silicon Bragg grating waveguide. We have given an alternative explanation of the effect via an indirect photonic transition. With this explanation the effect presented here represents a nonlinear analogue of signal trapping in a tapered plasmonic waveguide, when a surface plasmon polariton is not reflected but stopped inside the waveguide. In the case of the plasmonic waveguide the trapped light continuously increases its wavenumber keeping the spatial position constant. In the case of the moving front the light signal is trapped in the front and continuously changes its frequency and wavenumber.

To demonstrate the effect, we employed a CW signal and compared interactions in case of surfing, when signal and front have the same group velocities, and trapping, when slow signal light is caught by the fast front. In contrast to surfing, the trapping configuration could shift 20 times more energy from the CW signal. The shifted signal energy is expected to be compressed inside the front region. With sharper fronts and longer waveguides, a higher frequency shift and a larger energy accumulation can be envisaged. This opens a way for efficient one step pulse conversion in nonlinear waveguides. In addition, trapping effect can be used to realize a saturable-absorber-free pulsed laser in the mid-infrared wavelength from a CW laser. In this case large portions of the CW light will be cut out and compressed by the front without significant loss of average power.

The presented free-carrier front leads to intrinsic absorption of the signal interacting with the front. Alternatively, a refractive index perturbation induced by the Kerr effect can be employed. For silicon waveguide it would require an excitation with a MIR pulse.

## Supplementary Information

Below is the link to the electronic supplementary material.

Supplementary Material 1

## Data Availability

Data underlying the results presented in this paper may be obtained from the authors upon reasonable request.
