# Supplementary material for: Optical push broom effect by a moving refractive index front in a silicon Bragg waveguide
Source: Sci Rep. 2026 Jan 23;16:3050. doi: 10.1038/s41598-026-36302-x (PMC12830985; doi:10.1038/s41598-026-36302-x)
Supplement: Supplementary file 1 — Supplementary Material 1 [file 41598_2026_36302_MOESM1_ESM.docx]

Optical push broom effect by a moving refractive index front in a silicon Bragg waveguide

Boyi Zhang^1^, He Li^2^, Xinlun Cai^2^, Felix Vega^3^, Juntao Li^2^**^†^**, Manfred Eich^1,4^, Alexander Yu. Petrov^1,4†^, Mahmoud A. Gaafar^1,3,5*^

*^1^Institute of Optical and Electronic Materials, Hamburg University of Technology, Hamburg 21073, Germany*

*^2^State Key Laboratory of Optoelectronic Materials & Technology, Sun Yat-sen University, Guangzhou 510275, China*

*^3^Directed Energy Research Centre, Technology Innovation Institute, Abu Dhabi, SE45-01, United Arab Emirates*

*^4^Institute of Functional Materials for Sustainability, Helmholtz-Zentrum Hereon, Geesthacht 21502, Germany*

*^5^Department of Physics, Faculty of Science, Menoufia University, Menoufia, Egypt*

***^†^****lijt3@mail.sysu.edu.cn*

*^*^mahmoud.gaafar@tuhh.de*

Supporting Information

**Supplementary Note 1: Structure of the silicon Bragg grating waveguide (SBGW).** The employed SBGW has been fabricated on a Silicon-on-Insulator (SOI) wafer with a 220 nm thick silicon slab. The patterns were exposed on a spin-coated AR-P 6200.13 electron-beam photoresist by using the electron-beam lithography (EBL), which are then transferred into the silicon slab by using an inductively coupled plasma (ICP) dry etching. The residual photoresist is removed by the oxygen plasma etching and a microresist remover. The SBGW consist of a straight waveguide with periodic islands (wings) on both sides. Two grating couplers are used to couple the light inside the SBGW. To obtain the band gap at required wavelength, the grating waveguide has a width of 500 nm and the islands have the period of 324 nm. The height, width, and the thickness of the silicon wings are 220 nm, 220 nm and 190 nm, respectively, while the air gap between the wings and the straight waveguide is 35 nm. The straight waveguide is laterally tapered from 500 nm to 12 µm to within the 400 µm length at both ends of the SBGW to the width of the grating couplers. The length of the SBWG is 1 mm. An SEM image of the fabricated waveguide is shown in **Figure S1(a)**.

The fully etched grating couplers for efficient coupling between single mode fibers and silicon chips are fabricated in a single step of standard SOI processing, including EBL and ICP etching. The grating coupler is 20 µm long and 12 µm wide and consists of photonic crystal (PhC) columns with apodized hole sizes [1,2]. This coupler has a 3 dB bandwidth of 30 nm with a peak coupling wavelength of 1570 nm, and a minimal coupling loss of 7 dB per coupler when the coupling angle is 13$^{\circ}$. SEM of the grating coupler is shown in **Figure S1(b)**.


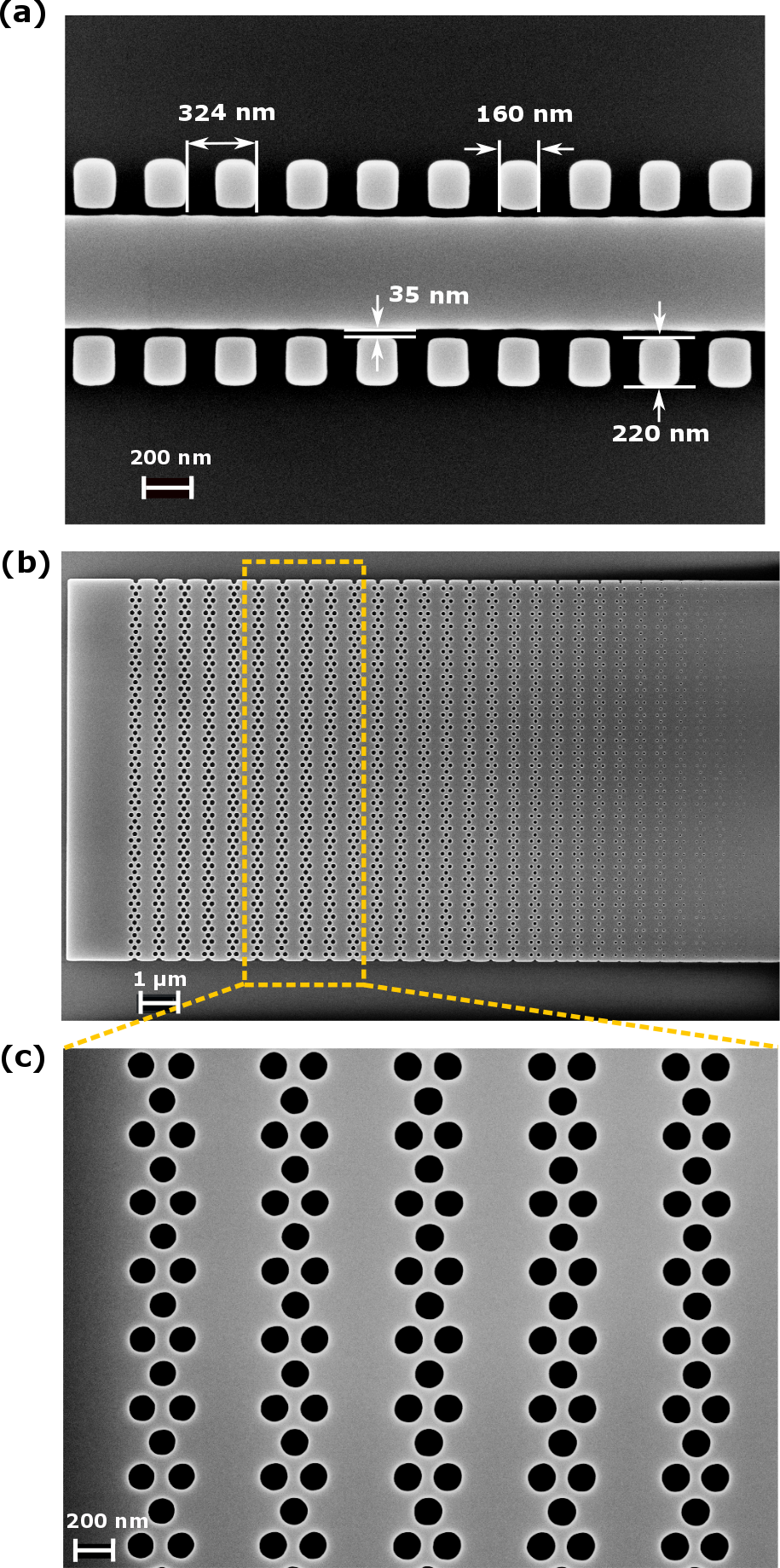


**Figure S1**. (a) SEM of the silicon Bragg grating waveguide. (b) SEM of the grating coupler.

**Supplementary Note 2: Original experimental spectra**

**Figure S2** shows the experimental output spectra at the output of our silicon Bragg grating waveguide for a transmitted CW signal wave’s power of (a) ≈ 4 μW at a wavelength of 1540 nm and (b) ≈ 2 μW at a wavelength of 1564.8 nm at a fixed pump peak power of 15 W. The spectrum of the CW signal that has not interacted with the pump pulses (red trace), of the pump pulses alone (orange trace), and of both the pump pulses and the signal (blue trace) are shown on a logarithmic scale in the upper graph. While they are subtracted on a linear scale (black trace) and displayed in the lower graph. We performed the subtraction on a linear scale in order to obtain only the converted signal without the residual of the CW input signal, in contrast to subtraction in log scale which would give us a ratio. The dashed line represents the center wavelength of the input signals. The signal spectra in the absence of the pump have components outside of CW line due to noise amplification in the EDFA amplifier. As we can see, in the presence of the pump pulse, clear peaks appear on both blue and red sides of the initial input signal wavelength in case of surfing, due to the positive and negative slope of the pump pulse, while the red frequency shift is negligible in case of trapping compared to the surfing case. We have to mention that the spectra of the signals without and with the pump have similar spectral features, which result from back reflections in the optical chip.


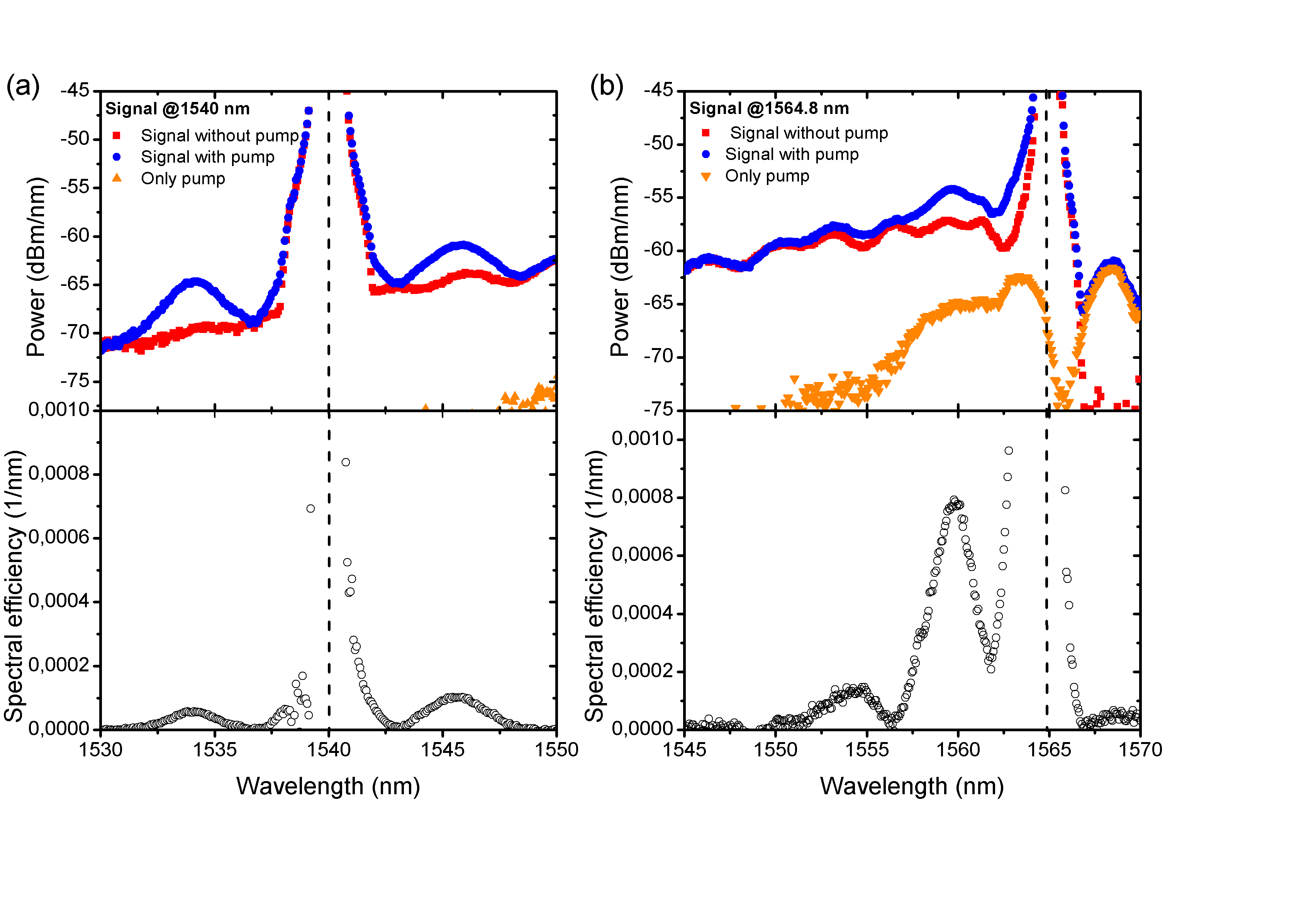


**Figure S2**: Experimental spectra recorded at the output of SBGW for an input signal at a wavelength of (a) 1540 nm and (b) 1564.8 nm and pump peak power of 15 W. The spectrum of the CW signal that has not interacted with the pump pulses (red trace), of the pump pulses alone (orange trace), and of both the pump pulses and the signal (blue trace) are shown on a logarithmic scale (upper graph). They are subtracted on a linear scale (black trace) in the lower graph. The dashed line represents the center wavelengths of the input signals light.

**Supplementary Note 3: Ray tracing calculations.** Complementing the theory and experiment, we performed ray tracing calculations of CW signal trapping inside the $1 mm$ long waveguide. We can split the CW signal into wave packets and track their trajectory in time and space separately. Such calculation is applicable to any waveguide with known dispersion and not limited to low contrast waveguides. It is established for fiber optics [3] and photonic crystal waveguides [4]. This approach is limited only by the temporal size of the wavepacket, similar as it is limited by the ray width in case of spatial rays. Namely the wavepacket in time should be much larger than the period of oscillation. Furthermore, it neglects the diffraction and interference effects between the wavepackets. Still it helps to predict the position of the trapped energy inside the front and explain the pulse compression effect.

In our simulation we start with a defined frequency of the wave packet and calculate its wavenumber and group velocity at the input. In a small time step a wave packet propagates a small distance according to its group velocity. Being inside the front the wave packet accumulates a frequency shift equal to $\Delta\omega=\partial\Delta\omega_{D}/\partial t\cdot\Delta t$ and a wavenumber shift according to $\Delta\beta=\Delta\omega/v_{f}$. This leads to a change of the group velocity which determines how the wave packet further propagates. Thus, we track the signal wave packets in space and time.

Here, we simulate the CW signal trapping inside the $1 mm$ long SBGW with parameters corresponding to experimental conditions. We assume that we can split the CW signal into wave packets located at different positions in the waveguide at a fixed point in time. We then launch these wave packets and follow their trajectories in a ray tracing approach.

We use light at 1.565 µm in a waveguide with a hyperbolic dispersion relation $\omega(\beta)=\omega_{\mathrm{PBG}}+{\Delta\omega}_{\mathrm{PBG}}\cdot\sqrt{1+\left[ {(\beta-\beta_{\mathrm{PBG}})}^{2}/{\Delta\beta}_{\mathrm{PBG}}^{2} \right]},$ emulating an upper band of a hyperbolic dispersion relation of SBGW, employing a PBG half opening of ${\Delta\omega}_{\mathrm{PBG}}= 2.198\cdot{10}^{12} 1/s$ $({\Delta f}_{\mathrm{PBG}}=0.35 \mathrm{THz}$) and a PBG center frequency $\omega_{\mathrm{PBG}}= 1.2\cdot{10}^{15} 1/s$ ($f_{\mathrm{PBG}}= 191.2 THz$). ${\Delta\beta}_{\mathrm{PBG}}={\Delta\omega}_{\mathrm{PBG}}/v_{g\infty}$ is the parameter that is chosen in such a way, that away from the band edge the dispersion relation converges to a straight line with group velocity $v_{g\infty}=c/4.1$, as shown in **Figure S1(a)**, where $\omega_{0}=\omega_{\mathrm{PBG}}+{\Delta\omega}_{\mathrm{PBG}}$. Here, $\beta_{\mathrm{PBG}}$ is the center wavenumber of the PBG, and $c$ is the velocity of light in vacuum. The band diagram shift induced by the front corresponding to both Kerr effect and FCs injections is described by the function ${\Delta\omega}_{D}\left( t \right)={{\Delta\omega}_{\mathrm{DFC}}}/2\cdot\left[ 1+\mathrm{erf} \left( \sqrt{4ln2}t/{t_{p}} \right) \right]-{\Delta\omega}_{\mathrm{DKerr}}\cdot exp\left[ {-4ln2t^{2}}/{{t_{p}}^{2}} \right]$, where $t_{p}=2$ ps is the temporal pump pulse width, ${\Delta\omega}_{\mathrm{DFC}}=1.38\cdot{10}^{12} 1/s$ (${\Delta f}_{\mathrm{DFC}}=0.22 \mathrm{THz}$) and${\Delta\omega}_{\mathrm{DKerr}}=0.69\cdot{10}^{12} 1/s$ (${\Delta f}_{\mathrm{DKerr}}=0.11 \mathrm{THz}$) are the maximum vertical band diagram shifts in frequency due to FCs injections and Kerr effect, respectively (see Supplementary Note 4). The input signal pulse is centered at frequency of 191.6 THz and has a velocity of $v_{g1}=c/14$, while the front has a velocity of $v_{f}=c/4.1$. The signal will be trapped and gradually frequency shifted along the phase continuity line (orange arrows in **Figure S3(a) and S3(c)**) as long as it stays inside the front.


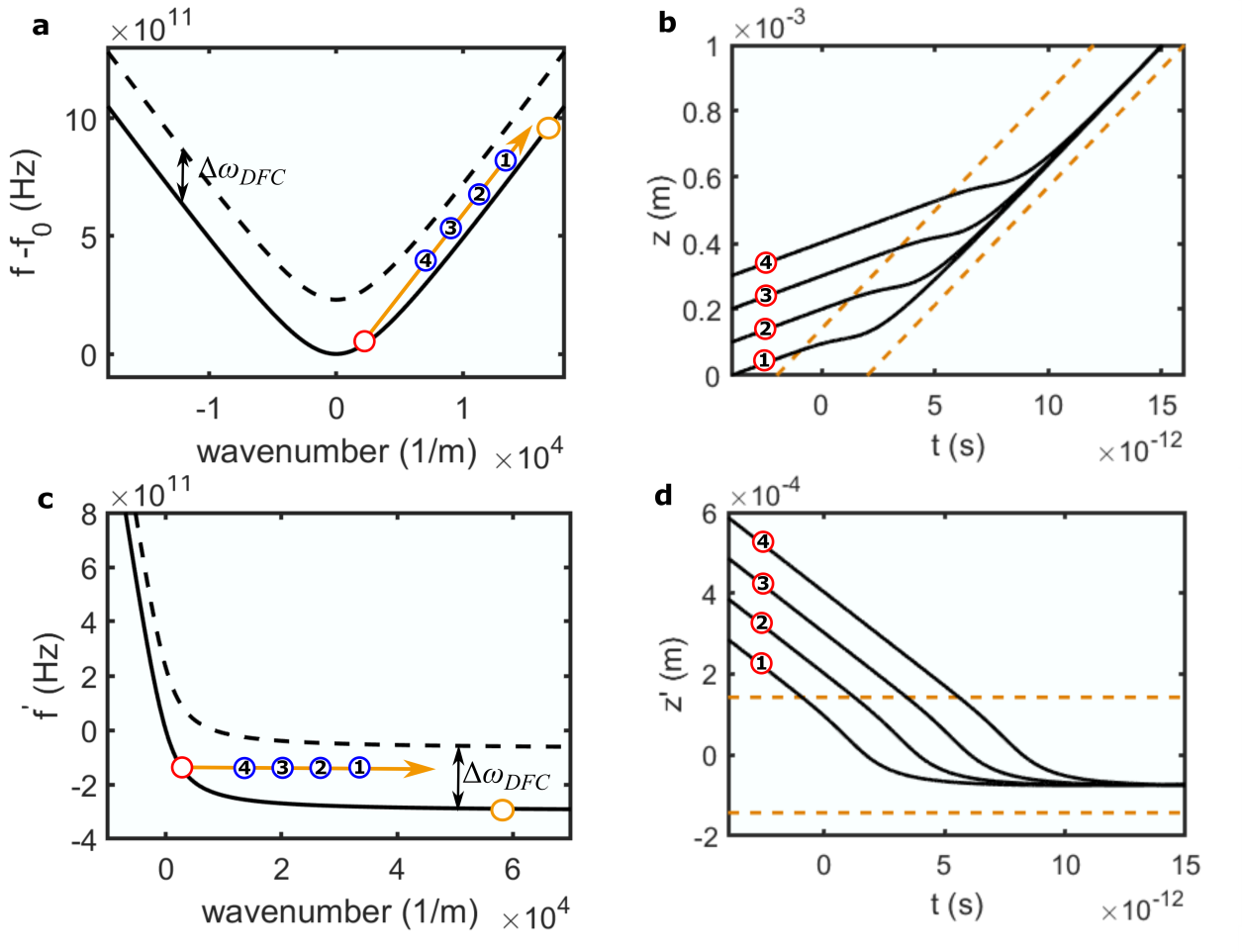


**Figure S3**: Simulation of the optical push broom effect inside a 1 mm long SBGW via ray tracing. (a) Original and shifted band diagram with a phase continuity line (orange line). The red and blue circles indicate the initial (slow mode) and final (fast mode) states of a signal wave, respectively. (b) The trajectories of four signal wave packets (black curves) represented in the stationary frame. The dashed orange lines represent the boundaries of $\pm t_{p}$. (c) The dispersion relation adapted for the frame moving with the front. (d) The trajectories of the signal in the frame moving with the front. The dashed horizontal orange lines represent the boundaries of $\pm z_{p}$, where $z_{p}=v_{f}\cdot t_{p}$ is the spatial pump width.

The simulation results of the optical push broom effect in the stationary (laboratory) frame are presented in **Figure S3(b)**. We consider the CW signal wave as being composed of individual wave packets located at different z-coordinate positions at a given point in time (they are denoted by the numbers 1-4). These wave packets then interact with the front with a delay, depending on their relative positions to the front at a given time. Their trajectories are shown by solid black lines. The dashed orange lines represent the boundaries of $\pm t_{p}$. Here, we show the trajectories of only four wave packets for clarity. Before the faster approaching index front encounters the wave packet, this wave packet moves along a straight line with constant group velocity of the signal $v_{g1}$, which corresponds to the slope of the original dispersion curve at the original frequency. In the beginning of the interaction the signal is gradually decelerated, due to the Kerr-induced positive slope, and then gradually accelerated until its velocity reaches that of the front. As can be seen, all wave packets of the same frequency converge to the same position inside the front.

The frequency change $\Delta\omega$ of the wave packet along the trajectory is proportional to the time spent inside the front $\Delta t$ (Equation 1) [5]. As different wave packets spend different time $\Delta t$ inside the front, they accumulate different frequency shifts, which leads to a broadening of the temporal and spatial frequency spectra (cf. several blue circles in **Figure. S3(a)** and **(c)**) and accordingly a narrowing in time and space of the output signal pulse, as shown in **Figure S3(b)**. The maximal wavelength shift is accumulated by the wave packet 1, which spends the longest time inside the front.

**Figure S3(c)** shows the dispersion relation adapted for the moving frame $\omega'\left( \beta\right)=\omega\left( \beta\right)-v_{f}\cdot(\beta-\beta_{0})$. In case of trapping it represents a saturating function with zero group velocity at infinite wavenumber $\beta$. In this corrected frame $z^{'}=z-v_{f}t$, the signal enters the standing perturbation and stops inside the front without reflection (**Figure S3(d)**). As we launch here a monochromatic signal it stops at a defined location inside the front where its group velocity becomes zero. In this frame the situation is very similar to light stopping in a tapered plasmonic waveguide, where group velocity of the signal also approaches zero in the taper [27-29].

**Supplementary Note 4: Free carrier generation and refractive index change**. The time dependence of the free carrier (FC) density $N\left( t \right)$ at a fixed point in space generated by two-photon absorption (TPA) of a pump pulse propagating in a silicon waveguide is described by [6]:

$\frac{dN(t)}{dt}=\frac{\beta}{2hf}\left( \frac{n_{g}}{n_{\mathrm{Si}}} \right)^{2}I^{2}\left( t \right)-\frac{N(t)}{\tau_{\mathrm{FC}}}$ **(S1)**

The ﬁrst term on the right side of this equation describes the generation of free carriers via the TPA process, while the second term describe the decay of the generated free carriers, characterized by the lifetime$\tau_{FC}$. Here,$h$ is Planck’s constant, $\beta=0.5 cm/GW$ is the TPA coefficient in silicon at a frequency $f=193.4 \mathrm{THz}$ [6], $n_{g}$ is the pump group index, $n_{\mathrm{Si}}$ is the silicon refractive index, and $I(t)$ is the pump pulse intensity.

The FC induced refractive index change can be described by the empirical relation presented by Soref et al. [7]:

${\Delta n}_{\mathrm{FC}}\left( t \right)=-8.8\cdot{10}^{-22}N\left( t \right)-8.5\cdot{10}^{-18}{N(t)}^{0.8}$ **(S2)**

While the Kerr induced refractive index change is described by:

${\Delta n}_{\mathrm{Kerr}}\left( t \right)=n_{2}\cdot I(t)$ **(S3)**

where $n_{2}$ is the nonlinear refractive index. Therefore, the total induced index change at the input of the waveguide can be described by:

${\Delta n}_{\mathrm{Total}}\left( t \right)={\Delta n}_{\mathrm{FC}}\left( t \right)+{\Delta n}_{\mathrm{Kerr}}(t)$ **(S4)**

We integrate the Equation S1 numerically assuming a Gaussian pulse intensity $I(t)=I_{0}\exp\left( {-4ln2\cdot t^{2}}/{t_{p}^{2}} \right)$, where $t_{p}$ is the pump pulse duration, and neglecting the FC recombination term as FCs have long life time in comparison to pulse duration.

The maximum of the generated FC density is given by:

$N_{FC, max}=\frac{\beta}{2hf}\left( \frac{n_{g}}{n_{\mathrm{Si}}} \right)^{2}I^{2}\sqrt{\pi}\frac{t_{p}}{\sqrt{8ln2}}$ **(S5)**

When a small perturbation of the refractive index is applied, the frequency of an eigenmode will change according to [8]:

$\Delta\omega=-\frac{\omega}{2}\frac{\int\Delta\epsilon(\vec{r)}\left| \vec{E}(\vec{r)} \right|^{2}d\vec{r}}{\int\epsilon(\vec{r)}\left| \vec{E}(\vec{r)} \right|^{2}d\vec{r}}$ **(S6)**

In our case most of the energy is guided in the waveguide and that is where the FCs are generated. Thus, we simplify the Equation S5 with the relation:

$\frac{\Delta\omega}{\omega}=-\frac{\Delta n}{n}$ **(S7)**

From this equation we can estimate the expected frequency shift of the optical mode according to the maximum refractive index change.

For $t_{p}=2 \mathrm{ps}$, $n_{g}=3.5$, a pulse peak power of ≈ 15 W at waveguide input, and by taking into account an effective mode area of the pump pulse of $0.1{\mu m}^{2}$ [9] and $n_{2}=9\cdot{10}^{-14} {\mathrm{cm}^{2}}/W$, we calculate the temporal index change at the input of our waveguide using Equation S4. Here the maximum generated FC density is $\approx9.5\cdot{10}^{17} 1/{\mathrm{cm}^{3}}$. The simulation results are presented in **Figure S4**. Orange, red, and black solid lines represent the index change due to free carrier injections ${\Delta n}_{FC}$, Kerr effect ${\Delta n}_{\mathrm{Kerr}}$, and the total index change ${\Delta n}_{\mathrm{Total}}$, respectively. We define the slope ${\partial\Delta n}/{\partial t}$ as the slope at a point of ${\partial^{2}\Delta n}/{\partial t^{2}}=0$ (green dashed line in **Figure S4**, which is in this case equal to ${{\Delta n}_{\mathrm{eff}}}/\tau$, where $\tau$ is the effective rise time of the negative front. $\tau$ is defined as the time from the maximum of the total index change ${\Delta n}_{\mathrm{Total}}$ till the crossing point of the green dashed line with the saturation line of ${\Delta n}_{\mathrm{Total}}$ (grey circles in **Figure S4**). According to Equation S7, this index change ${\Delta n}_{\mathrm{eff}}$ will lead to a shift of the dispersion relation $\Delta\lambda_{D}$, i.e. $\partial\Delta\lambda_{D}/\partial t=\Delta\lambda_{D}/\tau$. Therefore, the maximum possible wavelength shift of the signal in the structure $\Delta\lambda=\Delta\lambda_{D}\cdot\Delta t/\tau$, can be estimated by assuming that the maximal time spent inside the front $\Delta t$ is equal to the pump travelling time inside the 1 mm structure which is ≈ 12 ps, $\Delta\lambda_{D}=2.2 \mathrm{nm}$, and $\tau=1.9 \mathrm{ps}$. This yields to maximum wavelength shift of ≈ −13 nm, which approximately fits to the maximal obtained experimental shift of ≈ −11 nm in the experiment. We should also mention that the pump power decays in the waveguide due to absorption and scattering, and accordingly does the$\Delta n$. That can lead to a smaller accumulated frequency shift.


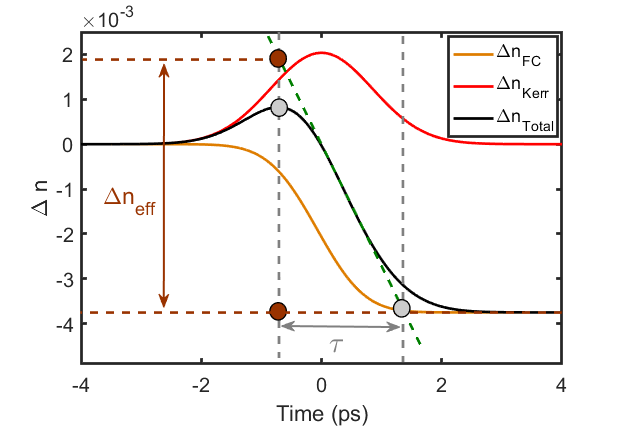


**Figure S4**: Simulation of the induced refractive index change as a function of time by a 2 ps-long pump pulse propagating in the silicon waveguide. Orange, red, and black solid lines represent the index change due to free carrier injections ${\Delta n}_{\mathrm{FC}}$, Kerr effect ${\Delta n}_{\mathrm{Kerr}}$, and the total index change${\Delta n}_{\mathrm{Total}}$, respectively. We define the slope ${\partial\Delta n}/{\partial t}$ as the slope at a point of ${\partial^{2}\Delta n}/{\partial t^{2}}=0$ (green dashed line), which is in this case equal to ${{\Delta n}_{\mathrm{eff}}}/\tau$, where $\tau$ is the effective rise time of the negative front.

References

1. Ding, Y., Ou, H. & Peucheret, C. Ultrahigh-efficiency apodized grating coupler using fully etched photonic crystals. *Opt. Lett.* **38,** 2732–2734 (2013).

2. Ding, Y., Peucheret, C., Ou, H. & Yvind, K. Fully etched apodized grating coupler on the SOI platform with −0.58  dB coupling efficiency. *Opt. Lett.* **39,** 5348–5350 (2014).

3. Philbin, T. G. *et al.* Fiber-Optical Analog of the Event Horizon. *Science* **319,** 1367 (2008).

4. Kondo, K. & Baba, T. Dynamic Wavelength Conversion in Copropagating Slow-Light Pulses. *Phys. Rev. Lett.* **112,** 223904 (2014).

5. Gaafar, M. A., Petrov, A. Y. & Eich, M. Free Carrier Front Induced Indirect Photonic Transitions: A New Paradigm for Frequency Manipulation on Chip. *ACS Photonics* **4,** 2751–2758 (2017).

6. Dekker, R. *et al.* Ultrafast Kerr-induced all-optical wavelength conversion in silicon waveguides using 1.55 μm femtosecond pulses. *Opt. Express* **14,** 8336–8346 (2006).

7. Soref, R. & Bennett, B. Electrooptical effects in silicon. *IEEE J. Quant. Electron.* **23,** 123–129 (1987).

8. J. D. Joannopoulos. *Photonic Crystals, Molding the Flow of Light* (Princeton University Press, 17–19 (2008)).

9. Li, J., O’Faolain, L., Rey, I. H. & Krauss, T. F. Four-wave mixing in photonic crystal waveguides: slow light enhancement and limitations. *Opt. Express* **19,** 4458–4463 (2011).
